# Qualitative transcriptional signatures for evaluating the maturity degree of pluripotent stem cell-derived cardiomyocytes

**DOI:** 10.1186/s13287-019-1205-1

**Published:** 2019-03-29

**Authors:** Rou Chen, Jun He, Yumei Wang, You Guo, Juan Zhang, Luying Peng, Duo Wang, Qin Lin, Jie Zhang, Zheng Guo, Li Li

**Affiliations:** 1 Key Laboratory of Arrythmias, Ministry of Education, Tongji University School of Medicine, Shanghai, China; 2 Medical Big Data and Bioinformatics Research Center, First Affiliated Hospital of Gannan Medical University, Ganzhou, Jiangxi China; 3 Fujian Key Laboratory of Medical Bioinformatics, Key Laboratory of Ministry of Education for Gastrointestinal Cancer, School of Basic Medical Sciences, Fujian Medical University, Fuzhou, Fujian China

**Keywords:** Pluripotent stem cell, Cardiomyocytes, Maturity score, Relative expression orderings

## Abstract

**Background:**

Pluripotent stem cell-derived cardiomyocytes (PSC-CMs) are widely used models for regenerative medicine and disease research. However, PSC-CMs are usually immature in morphology and functionality and the maturity of PSC-CMs could not be determined accurately. In order to reasonably interpret the experimental results obtained by PSC-CMs, it is necessary to evaluate the maturity of PSC-CMs and find the key genes related to maturation.

**Methods:**

Using the gene expression profiles of normal adult cardiac tissue and embryonic stem cell (ESC) samples, we identified gene pairs with identically relative expression orderings (REOs) within adult cardiac tissue but reversely identical in ESCs. Then, for a PSC-CM model, we calculated the maturity score as the percentage of these gene pairs that exhibit the same REOs in adult cardiac tissue. Lastly, the CellComp method was used to identify the maturation-related genes.

**Results:**

The maturity score increased gradually from 0.8401 for 18-week fetal cardiac tissue to 0.9997 for adult cardiac tissue. For four human PSC-CM models, the mature scores increased with prolonged culture time but were all below 0.8. The genes involved in energy metabolism, angiogenesis, immunity, and proliferation were dysregulated in the 1-year PSC-CMs compared with adult cardiac tissue.

**Conclusion:**

We proposed a qualitative transcriptional signature to score the maturity degree of PSC-CMs. This score can reasonably track the maturity of PSC-CMs and be used to compare different PSC-CM culture methods.

**Electronic supplementary material:**

The online version of this article (10.1186/s13287-019-1205-1) contains supplementary material, which is available to authorized users.

## Background

Pluripotent stem cells, including embryonic stem cells (ESCs) and induced pluripotent stem cells (iPSCs), have the potential to differentiate into any cell type including cardiomyocytes (CMs) [1]. Thus, pluripotent stem cell-derived cardiomyocytes (PSC-CMs) are widely used as models for drug screening in vitro [2] or for potential therapeutic applications in vivo [3]. However, the applications of PSC-CMs are limited due to the uncertainty of the maturation status [4–7]. Therefore, it is necessary to assess whether PSC-CMs are sufficiently mature in culture to make them suitable for their intended purpose.

To date, the maturity of CM is commonly evaluated by morphological and electrophysiological characteristics and cardiac-specific gene markers [8–10]. However, the morphological and electrophysiological characteristics are easily affected by subjective factors and thus difficult to quantify as strict maturity markers [11]. Also, there are many problems about cardiac-specific gene markers, such as MYH6, RYR2, KCNJ2, and cTnT [8, 12]. First, these markers are not exclusively expressed in cardiac myocytes. For example, cTnT is also expressed in non-cardiac cells such as smooth muscle cells [13–15]. Second, the cardiac-specific genes expressed in the early stage of heart development can also be reactive during a variety of pathophysiologic conditions, including hypoxia, ischemia, hypertrophy, atrophy, diabetes, and hypothyroidism [16–19]. Third, these markers cannot be used to evaluate the maturity degree of CM.

Given the abovementioned problems, several signatures based on the quantitative measurements of gene expression for evaluating CM maturity have been proposed [11, 20]. However, they usually could not be applied directly to other independent data because of experimental batch effects and data normalization biases [21–23]. In contrast, the within-sample relative expression orderings (REOs) of genes, which are qualitative transcriptional characteristics, are insensitive to experimental batch effects. Our previous researches have reported that REOs of gene pairs are highly stable in a type of tissues [24] or cells [25–27] in a particular status but tend to be widely disrupted in another status. Based on these unique advantages of REOs, Ao et al. used the hepatocellular carcinoma (HCC)-specific REO patterns of gene pairs to evaluate the similarity between HCC cell lines and HCC tissues [25]. Similarly, it should be possible to integrate heart-related data produced by different laboratories and select the adult cardiac tissue-specific and PSC-specific REO patterns of gene pairs to evaluate the similarity between PSC-CMs and adult cardiac tissue.

In this study, we identified a set of gene pairs with highly stable REOs within adult cardiac tissue but reversely stable REOs in ESCs. Then, for a PSC-CM model, we calculated the maturity score as the percentage of the selected gene pairs that exhibit the stable REOs in adult cardiac tissue. Using this scoring model to evaluate four human PSC-CMs, we found that the maturity scores of PSC-CMs were on the rise with the extension of culture time but were all below 0.8. Even when the culture time was extended to 1 year, the maturity of PSC-CMs reached 0.8274 but the rising degree was not obvious. Finally, using the REO-based CellComp method [27], we identified some key genes and functional pathways which could be responsible for the CM immaturity in vitro.

## Methods

### Data and preprocessing

All gene expression data analyzed in this study were downloaded from the GEO (http://www.ncbi.nlm.nih.gov/geo/) [28] and ArrayExpress (http://www.ebi.ac.uk/arrayexpress/) [29] repositories. We identified and screened the gene expression datasets by searching the following keyword: cardiac, heart, cardiomyocyte, embryonic stem cell, pluripotent stem cell, PSC-CMs, and ESC-CMs. Accordingly, a diverse set of cardiac-related gene expression data was collected comprising 17 datasets for human normal adult cardiac tissue, 41 datasets for human ESCs, 2 datasets for fetal cardiac tissue, and 4 datasets for PSC-CMs. The datasets for human normal adult cardiac tissue and ESCs samples were presented in Additional file 1: Table S1. The datasets for human PSC-CMs were described in Table 1. In Additional file 1: Table S2, we have briefly described the cell culture and isolation from tissue for each of the datasets according to the corresponding descriptions in the GEO or ArrayExpress repositories. The human cardiac samples took all the cells from the ventricle, atrium, or septum tissue and were approved by the ethics committee of hospital, local, or school.

**Table 1:** The datasets for PSC-CM from different culture times

Accession	Platform	Culture time (day)	Sample size	Reference
GSE35671	GPL6884	0, 3, 7, 10, 14, 20, 28, 35, 45, 60, 90, 120	36 (12 × 3)	Babiarz et al. [33]
GSE64189	GPL10558	0, 7, 14, 21, 28, 35, 42, 56	8 (8 × 1)	Zhang et al. [34]
GSE76523	GPL16791	0, 1, 2, 3, 4, 5, 6, 8, 10, 15, 31	11 (11 × 1)	Tompkins et al. [35]
GSE84815	GPL11154	0, 2, 5, 14, 30	9 (4 × 2 + 1 × 1)	Nakano et al. [36]

For the raw mRNA expression data (.CEL files) measured by the Affymetrix platform, the Robust Multi-array Average algorithm [30] was used to do background adjustment. For the data measured by the Illumina platform, we directly downloaded the processed data. Each probeset ID was mapped to Entrez gene ID according to the platform file. If a probeset was mapped to multiple or zero gene, then the data of this probeset was deleted. If multiple probesets were mapped to the same gene, the expression value for the gene was defined as the arithmetic mean of the value of multiple probesets. For GSE57338, we divided the 136 normal adult cardiac tissue samples into two parts according to the GSM series numbers of samples: the first 68 samples for establishing the highly stable gene pairs and the remaining 68 samples for evaluating the maturity.

### Identifying the gene pairs with stably reversal REOs between adult cardiac tissue and ESCs

For a particular tissue or cell line, pairwise comparisons were performed for all genes to identify gene pairs with stable expression ordering in accumulated samples from different data sources. For each gene pair (G_i_, G_j_), being viewed as an event with only two possible outcomes (G_i_ > G_j_ or G_i_ < G_j_), the gene pairs that the expression level of G_i_ was higher (or lower) than that of G_j_ in all accumulated adult cardiac tissue or ESCs samples were defined as highly stable gene pair.

A stable reversal gene pair (G_i_ and G_j_) was selected when its REO, G_i_ > G_j_ or G_i_ < G_j_ in expression level, was identical in all adult cardiac tissue samples and was reversed (G_i_ < G_j_ or G_i_ > G_j_) in all ESCs samples.

### Maturity score

The REOs of stably reversal gene pairs in adult cardiac tissue samples were treated as the golden standard. The maturity score in every PSC-CMs sample was calculated as *k*/*n*, where *n* was the number of the stably reversal gene pairs and *k* was the number of reversal gene pairs with the consistent REOs in the PSC-CMs and adult cardiac tissue. If the REOs of the stable reversal gene pairs in PSC-CMs are closer to that of adult cardiac tissue, the maturity score is closer to 1. For PSC-CMs with two or three technical replicates, we calculated the average maturity score of these PSC-CMs as the final maturity score.

### CellComp algorithm

For small-scale cell line data commonly with only two or three technical replicates, the traditional methods such as SAM, RP, and FC lack statistical power or statistical control for differential expression analysis [27]. Thus, we used the REO-based CellComp algorithm which has higher statistical power for small-scale data sets to identify differentially expressed genes (DEGs).

In our study, we adjusted the reference for the highly stable gene pairs from accumulated normal cardiac tissue samples. Then, based on the Fisher exact test, DEGs were identified in PSC-CMs through finding genes with large expression changes that may lead to the stably reversal REOs in two or three PSC-CM replicates compared to the reference, as described in detail in our previous work [27]. The Benjamini–Hochberg method was used to control false discovery rate (FDR) in the multiple tests. The CellComp algorithms are freely available online at https://github.com/pathint/reoa.

### Functional enrichment analysis

Functional enrichment analyses were performed based on Gene Ontology (GO) [31]. First, we used the CellComp algorithm to identify DEGs. Then, the Go-function algorithm [32] was adopted to identify the GO annotations by the following categories: biological processes, molecular functions, and cellular components. The Benjamini–Hochberg method was adopted to estimate the FDR.

## Results

### The REO-based maturity score

We collected 195 human normal adult cardiac tissue and 116 human ESCs (hESCs) samples from 57 datasets measured by ten platforms (see Additional file 1: Table S1). Then, we identified 16,576,526 and 30,068,382 gene pairs with identical REOs in all adult cardiac tissue and all hESCs samples, respectively. Among these gene pairs, 223,004 gene pairs had reversal REOs between adult cardiac tissue and hESCs. Based on the REOs of 223,004 gene pairs in adult cardiac tissue, we calculated the maturity score of every PSC-CM sample as the percentage of theses gene pairs that showed the same REOs in adult cardiac tissue (see the “Methods” section).

Firstly, we evaluated the maturity degree of adult cardiac tissue and hESCs. The average maturity score of 68 normal adult cardiac tissue samples from GSE57338 was 0.9997. Conversely, the average maturity score of 72 hESCs samples from GSE38662 was 0.0004 (see Additional file 1: Table S3). Similarly, we evaluated the fetal cardiac tissue samples from two datasets and found the maturity score gradually increased from 0.8401 to 0.9665 with the prolonged gestation from 18 to 39 weeks, as showed in detail in Table 2. These results demonstrated that this score can reasonably evaluate the maturity of the cardiac tissue.

**Table 2:** The maturity scores of fetal cardiac tissue in different gestational ages

Accession	Platform	Gestational age (week)	Maturity score
GSE50704	GPL6884	18	0.8401
		20^a^	0.8425
		20^b^	0.8627
E-MEXP-2654	A-AFFY-141	33	0.9444
		39	0.9665

^a,b^They were obtained from two different fetal tissue samples

**Fig 1:**
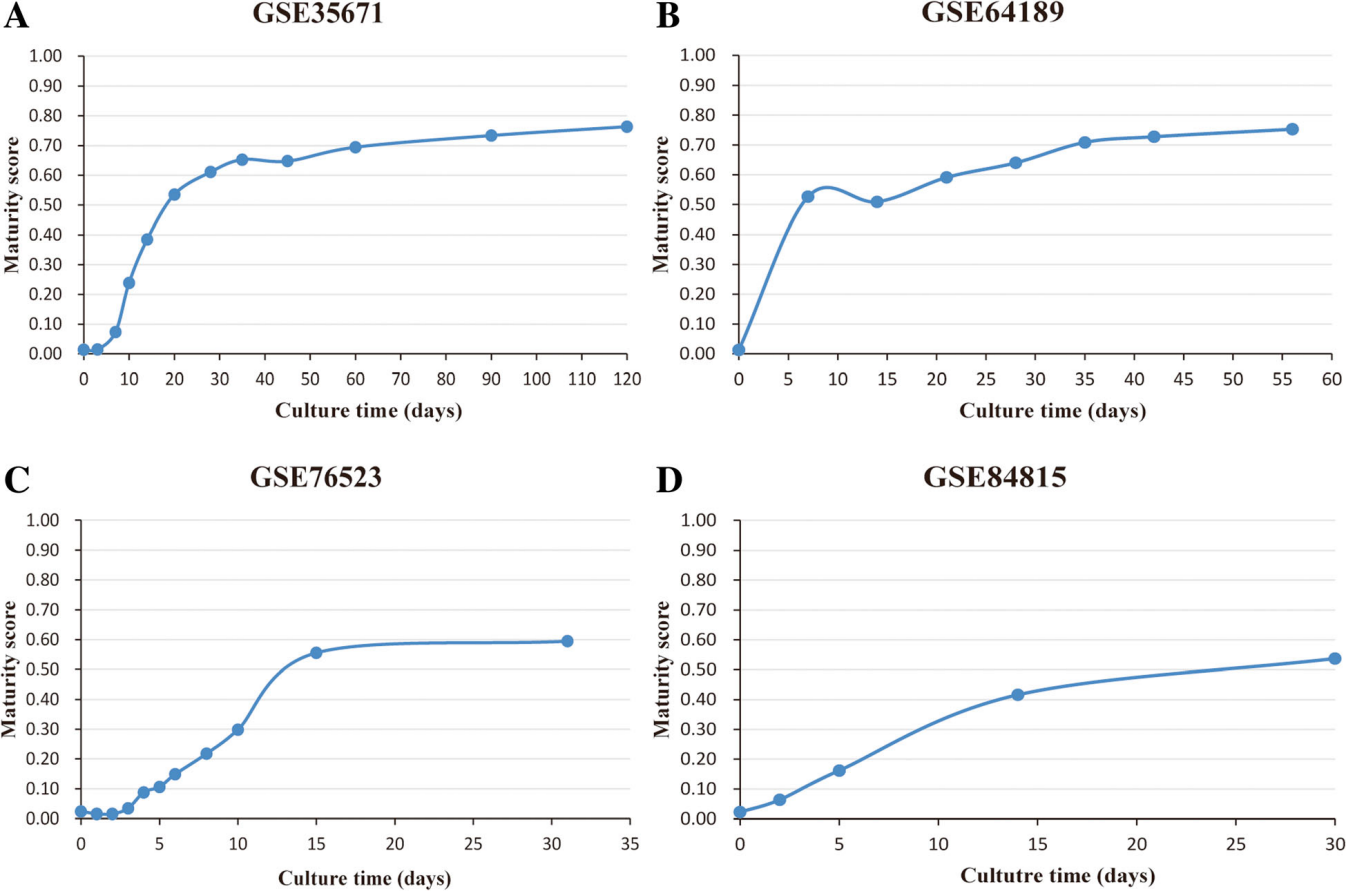
The maturity score of four human PSC-CMs from different culture time points

Then, we used four human PSC-CM datasets generated by four experimental methods [33–36] to evaluate the maturity degree of PSC-CMs at different culture time points (Table 1). The result showed that, for each of the four experiments, the maturity score was on the rise with prolonged culture time (Fig. 1). The maturity scores of the four PSC-CMs, which were cultured for 30, 31, 56, and 120 days, reached 0.5371, 0.5943, 0.7529, and 0.7638, respectively. Notably, the maturity scores of PSC-CMs from different sources were all below 0.8. Recent studies reported that long-term cultures may enhance CM maturation [8, 37, 38]. However, for human PSC-CMs cultured for 1 year (GSE62913), the maturity score was only 0.8274 and the rising degree of maturity was not obvious.

The above results indicated that current differentiation protocols in vitro could not induce mature CMs. Thus, there must be some key molecules that are aberrant in the regulation of CM maturation.

### The key genes and pathways influencing the maturation of PSC-CMs

Taking 195 human adult cardiac tissue samples as the reference, we applied the REO-based CellComp method [27] to identify DEGs in PSC-CMs compared to adult cardiac tissue (FDR < 0.05). The CellComp is an efficient tool for analyzing small-scale cell line data with only two or three technical replicates, and it can identify DEGs whose change can disrupt the rank or correlation structure of the transcriptome.

In the 1-year PSC-CMs with three technical replicates (GSE62913), 1323 DEGs (886 up- and 437 downregulated) were detected by the CellComp method. Functional enrichment analysis (FDR < 0.05, Additional file 1: Table S4) showed that the downregulated genes were significantly enriched in the circulatory system, angiogenesis, metabolism, and immune system-related pathways, whereas the upregulated genes were enriched in cell proliferation-related pathways such as nuclear division and sister chromatid segregation.

The top five DEGs, participating in the largest numbers of gene pairs with reversal REOs between the ESCs and the adult cardiac tissue, were showed in Table 3. The five genes were all downregulated in 1-year PSC-CMs relative to adult CMs. It implied that the aberrant expression of these genes could induce great variation of transcriptome structure which can retard the transformation of PSC-CMs from an immature state to mature state. CASQ2, participating in 4987 reversal gene pairs, can facilitate the maturation of Ca^2+^ handling by modulating RyR activities to coordinate the rates of sarcoplasmic reticulum Ca^2+^ release and load, which is crucial for CM maturation [39]. CKM is related with mitochondrial functions, including intracellular energy transport and ATP generation, which can promote the oxidative metabolism [40, 41]. It has been reported that, as the CMs mature, mitochondrial oxidative metabolism increases with fatty acid oxidation, providing 90% of the heart’s energy demands [12]. CD36, a cell adhesion molecule, can regulate fatty acid transport [42]. Cell adhesion molecules are related with intercellular communication. Studies pointed out that the cell–cell interaction increasing would promote CM maturation [43, 44]. CMYA5, a Z-disc-related protein, involved in the construction of myocardial fiber [45, 46]. The overexpression of CMYA5 is associated with left ventricular hypertrophy [47, 48]. DCN, a structural constituent of the extracellular matrix, plays roles in proliferation, autophagy, angiogenesis, and fibrin organization [49, 50], and its overexpression can facilitate the formation of blood vessels, which is important for the formation of highly vascularized adult cardiac tissue [51, 52].

**Table 3:** The top five DEGs participating in the largest numbers of gene pairs with reversal REOs in ESCs compared with the mature adult CMs

Gene symbol	Gene ID	The number of gene pairs	Possible function
CASQ2	845	4987	Calcium treatment
CKM	1158	4712	Intracellular energy transport, muscle contraction, and ATP generation
CD36	948	3584	Cell adhesion molecule and regulating fatty acid transport
CMYA5	202,333	3177	Structure and contraction
DCN	1634	3043	Angiogenesis, autophagy, inflammation, and tumorigenesis

The above results indicated that these genes and pathways may be important factors for the CM maturation in the late stage of myocardial differentiation.

## Discussion

In this study, we established a REOs-based score method to assess the maturity degree of PSC-CMs. It is highly robust against large measurement variations introduced by experimental batch effects and platform differences. Essentially, the maturity scores are 1 for adult cardiac tissues and 0 for ESCs, and the scores for the human PSC-CMs increase with the extension of culture time. These results suggested that this score can reasonably evaluate the maturity degree of human PSC-CMs.

Our analysis showed that the maturity of PSC-CMs in vitro can hardly exceed 0.8. Even when PSC-CMs were cultured for 1 year, the maturity score was just 0.8274. Thus, we screened the aberrantly expressed genes in 1-year PSC-CMs compared with adult cardiac tissue samples. The genes involved in energy metabolism, proliferation, angiogenesis, and immunity were dysregulated. We identified some DEGs such as CASQ2, CKM, CD36, CMYA5, and DCN that participated in largest numbers of reversal gene pairs, which could induce the great variation of transcriptome correlation structure and retard the transformation of PSC-CMs from an immature state to mature state. These DEGs might be of special biological significance for CM maturation, because the functionally related genes tend to express coordinately in a stable state but widely altered in another state [53]. Further studies are necessary to determine their roles for PSC-CM maturation.

Many approaches have been reported to improve the maturation of PSC-CMs [43, 54–56]. However, there are no definitive markers available to determine which method can better promote the CM maturation. The maturity score can be used to compare different culture methods. For example, the maturity scores for three human PSC-CMs cultured for 14 days from protocol 1 (GSE35671), protocol 2 (GSE64189), and protocol 3 (GSE84815) were 0.3811, 0.5087, and 0.4210, respectively. Moreover, the maturity score of 56-day PSC-CMs from protocol 2 (0.7529) was higher than that of 90-day PSC-CMs from protocol 1 (0.7339). The maturity score of 21-day PSC-CMs from protocol 2 (0.5909) was close to that of 31-day PSC-CMs from protocol 3 (0.5943). Therefore, we can infer that protocol 2 may be more effective to promote CM maturation than protocols 1 and 3.

## Conclusion

In summary, we identified a qualitative transcriptional signature which can reasonably track the maturity of PSC-CMs. As a score, it can be used to compare different culture methods for PSC-CM maturation. By REOs-based method, we further identified some genes and pathways which may be important factors for the CM maturation in the late stage of myocardial differentiation.

## Additional file

Additional file 1: **Table S1.** The datasets for human normal adult cardiac tissue and ESCs samples. **Table S2.** The brief description of the cell culture and isolation from cardiac tissue for the datasets. **Table S3.** The maturity scores for 68 normal adult cardiac tissue and 72 ESC samples. **Table S4.** The enriched pathways of DEGs for 1-year PSC-CMs. (XLSX 26 kb)

## Abbreviations

CMs: Cardiomyocytes
DEGs: Differentially expressed genes
ESCs: Embryonic stem cells
FDR: False discovery rate
HCC: Hepatocellular carcinoma
iPSCs: Induced pluripotent stem cells
PSC-CMs: Pluripotent stem cell-derived cardiomyocytes
REOs: Relative expression orderings
